# Recent progress on the interaction between insects and *Bacillus thuringiensis* crops

**DOI:** 10.1098/rstb.2018.0316

**Published:** 2019-01-14

**Authors:** Yutao Xiao, Kongming Wu

**Affiliations:** 1 Agricultural Genomics Institute at Shenzhen, Chinese Academy of Agricultural Sciences, Shenzhen 518120, People's Republic of China; 2 The State Key Laboratory for Biology of Plant Disease and Insect Pests, Institute of Plant Protection, Chinese Academy of Agricultural Sciences, West Yuanmingyuan Road, Beijing 100193, People's Republic of China

**Keywords:** Bt crops, pest, transgenic technology, resistance

## Abstract

Extensive use of chemical pesticides poses a great threat to the environment and food safety. The discovery of *Bacillus thuringiensis* (Bt) toxins with effective insecticidal activity against pests and the development of transgenic technology of plants opened a new era of pest control. Transgenic Bt crops, including maize, cotton and soya bean, have now been produced and commercialized to protect against about 30 major coleopteran and lepidopteran pests, greatly benefiting the environment and the economy. However, with the long-term cultivation of Bt crops, some target pests have gradually developed resistance. Numerous studies have indicated that mutations in genes for toxins activation, toxin-binding and insect immunization are important sources in Bt resistance. An in-depth exploration of the corresponding Bt-resistance mechanisms will aid in the design of new strategies to prevent and control pests. Future research will focus on Bt crops expressing new genes and multiple genes to control a broader range of pests as part of an integrated pest management programme.

This article is part of the theme issue ‘Biotic signalling sheds light on smart pest management’.

## 1. Introduction

Since the beginning of agricultural society, pest management has been an important part of agricultural production. The advent of various chemical pesticides has promoted crop production and been the main pest control measure [1]. Chemical pesticides have also brought serious problems such as the emergence of insect resistance, the re-emergence of insect pests, threats to non-target organisms, soil contamination, environmental pollution, ecological hazards and food safety problems [2–4]. With the continuous improvement of living standards, pollution and food safety problems caused by chemical pesticides have brought widespread demands for more effective and safe pest control technology [5].

*Bacillus thuringiensis* (Bt), a Gram-positive soil bacterium, produces endospores and a poisonous parasporal crystal. After ingestion by a herbivorous insect, the crystal dissolves in the alkaline environment of the insect midgut, releasing one or more insecticidal crystalline proteins (ICPs), also known as a delta-endotoxin [6]. ICPs can be activated by midgut proteases. Once activated, the ICPs interact with larval midgut epithelial cells and destroy membrane integrity, ultimately leading to insect death [7,8].

As a biogenic insecticide, the Bt-ICP has significant advantages over chemical insecticides [9], but direct spraying of Bt has many problems. Poor product stability, easy inactivation under visible light, short residual effect period, slow speed of killing, and susceptibility to soil and environmental factors have severely limited commercialization of Bt insecticides. As more Bt genes have been discovered and transgenic technology has improved, generating Bt crops has become more convenient. Since the first Bt-insecticidal crystalline protein gene was cloned and sequenced in 1981, 993 Bt toxin-encoding genes have been cloned and classified, including 801 *Cry* genes, 40 *Cyt* genes and 152 *Vip* genes (http://www.lifesci.sussex.ac.uk/home/Neil_Crickmore/Bt/), providing abundant material for producing transgenic Bt crops. In the past 22 years, transgenic Bt crops have been widely developed and grown commercially, contributing greatly to the control of numerous agricultural pests [10].

With long-time use of Bt crops, however, target pests can respond actively by evolving resistance to the crop as in the case of *Busseola fusca*, *Diabrotica virgifera virgifera*, *Helicoverpa zea*, *Pectinophora gossypiella* and *Spodoptera frugiperda* [11]. By understanding the mechanism underlying this resistance in target pests, we hope to devise strategies to delay resistance evolution. In addition, crops expressing novel Bt toxins have been developed and popularized. In this paper, we summarize the Bt-transgenic crops that have been commercialized and the target pests and recent progress on the interaction between insects and Bt crops, the mechanisms underlying resistance to Bt toxins, and strategies to avoid the emergence of resistance.

## 2. Commercialized *Bacillus thuringiensis* crops and the target pests

Genetically modified (GM) plants producing Bt genes have been approved for commercialized cultivation in most of the major grain and economic crops, including maize, cotton, soya bean, rice, potato, brinjal, tomato and sugarcane. Currently, Bt crops are mainly cultivated to manage coleopteran, lepidopteran and some hemipteran insects (table 1).

**Table 1.** Bt crops and their target pests.

crop	Bt toxin	commercialized (yes/no)	target pest	reference
maize	Cry1Ab	yes	*Ostrinia furnacalis*	[12]
			*Ostrinia nubilalis*	[13]
			*Spodoptera frugiperda*	[14]
			*Busseola fusca*	[15]
			*Diatraea saccharalis*	[16]
			*Diatraea grandiosella*	[17]
			*Chilo partellus*	[18]
	Cry1Ac	yes	*Ostrinia furnacalis*	[12]
			*Chilo partellus*	[18]
			*Spodoptera frugiperda*	[14]
	Cry1Fa2	yes	*Ostrinia nubilalis*	[13]
			*Spodoptera frugiperda*	[19]
	Cry1F	yes	*Spodoptera frugiperda*	[20]
			*Diatraea saccharalis*	[20]
			*Diatraea grandiosella*	[20]
			*Ostrinia nubilalis*	[21]
	Cry9C	yes	*Ostrinia nubilalis*	[22]
	Cry1A.105	yes	*Spodoptera frugiperda*	[20]
			*Diatraea saccharalis*	[20]
			*Diatraea grandiosella*	[20]
	Cry2Ab2	yes	*Spodoptera frugiperda*	[20]
			*Diatraea saccharalis*	[20]
			*Diatraea grandiosella*	[20]
	Vip3Aa20	yes	*Spodoptera frugiperda*	[23]
	Cry3Bb1	yes	*Diabrotica virgifera virgifera*	[24]
	Cry34Ab1	yes	*Diabrotica virgifera virgifera*	[24]
	Cry35Ab1	yes	*Diabrotica virgifera virgifera*	[24]
	mCry3A	yes	*Diabrotica virgifera virgifera*	[25]
	eCry3.1Ab	yes	*Diabrotica virgifera virgifera*	[25]
	Cry1Ie	no	*Ostrinia furnacalis*	[26]
			*Helicoverpa armigera*	[26]
	Cry1C	no	*Ostrinia furnacalis*	[27]
cotton	Cry1Ac	yes	*Helicoverpa armigera*	[28]
			*Heliothis virescens*	[29]
			*Pectinophora gossypiella*	[30]
			*Helicoverpa zea*	[28]
			*Helicoverpa punctigera*	[31]
			*Spodoptera exigua*	[32]
			*Trichoplusia ni*	[33]
	Cry2Ab2	yes	*Helicoverpa armigera*	[28]
			*Helicoverpa punctigera*	[31]
			*Trichoplusia ni*	[33]
			*Heliothis virescens*	[29]
			*Pectinophora gossypiella*	[30]
			*Helicoverpa zea*	[29]
			*Spodoptera exigua*	[34]
	Vip3A(a)	yes	*Helicoverpa armigera*	[28]
			*Heliothis virescens*	[29]
			*Helicoverpa zea*	[29]
			*Helicoverpa punctigera*	[35]
	Cry1F	yes	*Helicoverpa armigera*	[28]
			*Helicoverpa zea*	[36]
	Cry1Ab	yes	*Helicoverpa armigera*	[28]
			*Heliothis virescens*	[37]
			*Helicoverpa zea*	[37]
	Cry2Ae	yes	*Helicoverpa armigera*	[28]
			*Heliothis virescens*	[37]
			*Helicoverpa zea*	[37]
	Cry1Ca	no	*Spodoptera exigua*	[38]
	Cry51Aa	no	*Lygus hesperus*	[39]
	Cry15Aa	no	*Apolygus lucorum*	[40]
rice	Cry1Ab	no	*Chilo suppressalis*	[41]
			*Cnaphalocrocis medinalis*	[41]
			*Scirpophaga incertulas*	[42]
	Cry1Ac	no	*Chilo suppressalis*	[41]
			*Cnaphalocrocis medinalis*	[41]
			*Scirpophaga incertulas*	[42]
	Cry1C	no	*Chilo suppressalis*	[43]
			*Cnaphalocrocis medinalis*	[44]
	Cry2A	no	*Chilo suppressalis*	[45]
			*Cnaphalocrocis medinalis*	[46]
			*Scirpophaga incertulas*	[46]
	Cry9C	no	*Chilo suppressalis*	[45]
	Vip3H	no	*Scirpophaga incertulas*	[47]
			*Chilo suppressalis*	[47]
	Cry64Ba	no	*Laodelphax striatellus*	[48]
			*Sogatella furcifera*	[48]
	Cry64Ca	no	*Laodelphax striatellus*	[48]
			*Sogatella furcifera*	[48]
potato	Cry3A	yes	*Leptinotarsa decemlineata*	[49]
	Cry1Ab	no	*Phthorimaea opercullela*	[50]
soya bean	Cry1Ac	yes	*Spodoptera litura*	[51]
			*Anticarsia gemmatalis*	[52]
brinjaul	Cry1Ac	yes	*Leucinodes orbonalis*	[53]
sugarcane	Cry1Ab	yes	*Diatraea saccharalis*	[54]

### (a) *Bacillus thuringiensis* maize

The early Bt genes inserted into maize were primarily *Cry1Ab*, *Cry1Ac* and *Cry2A* [55]. Subsequently, *Cry34/35Ab1* and *Cry3Bb1* genes were introduced to control closely related pest species [56,57]. In recent years, Bt genes have started to be stacked in GM maize. For example, a maize variety expressing five Bt genes (*eCry3.1Ab*, *mCry3A*, *Cry1Ab*, *Cry1Fa2* and *Vip3Aa20*) was planted commercially in 2013, and another GM maize expressing six Bt genes (*Cry2Ab2*, *Cry1A.105*, *Cry1F*, *Cry34Ab1*, *Cry35Ab1* and *Cry3Bb1*) was introduced in 2017. At present, even more GM maize varieties that produce multiple Bt genes have been approved (http://www.isaaa.org/gmapprovaldatabase/default.asp). *Chilo partellus* caused much less damage to the leaves of three Bt maize hybrids producing Cry1Ab than to those of non-Bt iso-hybrids, and the mortality of *C. partellus* larvae feeding on Bt maize was 79.4–100% in laboratory tests [58]. Cry3Bb1 is one of the most commonly used Bt toxins in GM maize, has good insecticidal activity against Colorado potato beetle (*Leptinotarsa decemlineata*) and even better activity against western corn rootworm (*D. v. virgifera*) [59,60]. In eastern North Dakota (United States), the total feeding injury and population level of western corn rootworm were the lowest on Cry3Bb1+Cry34/35Ab1 hybrids than on Bt maize producing either Cry3Bb1 or Cry34/35Ab1 protein alone [61].

### (b) *Bacillus thuringiensis* cotton

In 1996, Bt cotton producing Cry1Ac was first released for cultivation in Australia and the United States, and in China the next year [11,62]. Early in the history of GM cotton, Bt cotton expressing Cry1Ac gave good control of major target pests such as cotton bollworm (*Helicoverpa armigera*) and pink bollworm (*P. gossypiella*), and obviously, the population of the target pests decreased [63]. However, long-term planting of a cotton variety with one Bt gene brings the risk of resistance, so two-toxin cotton, including different toxin combinations of Cry1Ac+Cry2Ae, Cry1Ab+Vip3A(a), Cry1Ab+Cry2Ab2, Cry1Ab+Cry2Ae and Cry1Ac+Cry2Ab2, began to be studied and tested at the end of the twentieth century. In many countries, Bt cotton expressing *Cry1Ab*+*Cry2Ab2* gradually replaced GM cotton expressing a single-Bt gene [62]. When control efficiency was monitored, the number of cotton bollworm larvae at the second, third and fourth generations on two-toxin cotton was 81.4%, 87.1% and 87.0%, respectively, lower than on non-Bt cotton. Compared with one-toxin cotton, the number of larvae decreased by 11.1%, 33.3% and 57.1%, respectively [64]. Some three-toxin Bt cottons that express Cry1Ac+Cry1F+Vip3A(a), Cry1Ac+Cry2Ab+Vip3A(a), and Cry1Ab+Cry2Ae+Vip3A(a) have also been developed (http://www.isaaa.org/gmapprovaldatabase/gmtrait/default.asp?TraitID=6&GMTrait=Lepidopteran%20insect%20resistance). Although cotton bollworm and other lepidopteran pests have been well controlled, the non-target pest, the mirid bug (*Lygus hesperus*; Hemiptera: Miridae), has emerged as the main pest [65]. Interestingly, Monsanto has found that Cry51Aa2 had insecticidal activity against mirid nymphs. Cry51Aa2 belongs to the Mtx (mosquitocidal toxins) group of proteins, which differs structurally from the widely used Cry1A [39,66] and is also insecticidal against *Apolygus lucorum* [41]. At present, mirid is mainly controlled by chemical pesticides. If the *Cry51Aa2* gene can be co-introduced into cotton with another Bt toxin gene(s), the risk of mirids outbreak and environmental problems from pesticides should be greatly lowered.

### (c) *Bacillus thuringiensis* soya bean

GM soya bean accounts for the largest proportion of all GM crops planted, so far, Cry1Ac, Cry1F, Cry1A105 and Cry2Ab2 have been studied in soya bean. Monsanto developed a GM soya bean variety MON87701 (expressing Cry1Ac) and MON89788 (expressing 5-enolpyruvylshikimate-3-phosphate synthase (EPSPS)) and was first commercially released in Brazil during the 2013–2014 growing season [10]. Bt soya bean varieties MON87701 and MON87701RR2Y (expressing Cry1Ac+EPSPS) were significantly resistant to *H. armigera* throughout the whole growing season when first released; *H. armigera* larvae had a survival rate between 5.4% and 24.4%, significantly lower than after feeding on non-Bt leaves (71–94.9%). The survival rate, larval mass and female fecundity of *Spodoptera litura* also significantly decreased when Bt soya bean was planted [51]. Soya bean MON87701×MON89788 also has a high preventive effect against *Heliothis virescens* [67]. In an efficacy test with a modified Bt soya bean cultivar, 100% mortality of *H. armigera* was obtained for all six instars [68].

### (d) *Bacillus thuringiensis* rice

Since Fujimoto first introduced *Cry1Ab* into a japonica rice variety, several other Bt rice materials with good insect resistance have been developed. T1C-19 and T2A-1 are two widely used Bt rice lines, which have insecticidal activity in various insect tissues and organs and confer resistance during different reproductive periods [69,70]. Because deploying two or more Bt genes in one rice variety can delay the emergence of pest resistance [71], Cheng *et al*. [72] introduced the *Cry1Ab*/*Cry1Ac* fusion gene into various rice plants and obtained highly efficient expression strains. Field experiments with rice strain Minghui63 (*Cry1Ab*+*Cry1Ac* fusion gene) and its hybrid strain Bt-Shanyou63 showed high resistance against target pests [42]. The incidence of *Chilo suppressalis* larvae on another variety Huahui1, which also expresses *Cry1Ab/Cry1Ac*, was significantly reduced by 84.9–100% [73]. In addition, the incidence of dead heart/white head plants and damaged plants caused by *C. suppressalis* on Bt rice was significantly lower (30.8–98.3% and 11.4–96.6%, respectively) than on the control variety. Recently, Cry64Ba and Cry64Ca proved to be effective in controlling rice planthoppers, thus providing a novel strategy to manage hemipteran pests [48].

### (e) Other *Bacillus thuringiensis* crops

GM potato expressing Cry3A showed significant resistance to Colorado potato beetle [49]. Potato producing Cry1Ab was also effective against potato tuber moth (*Phthorimaea operculella*), and transgenic tubers caused significant growth retardation and high mortality of neonatal tuber moth larvae [50]. In 2014, Bangladesh began to plant *Cry1Ac*-transgenic brinjal to control the main pest *Leucinodes orbonalis* [10]. With the continuous improvement of GM technology, more potential Bt genes will be discovered and applied in GM crops. In general, Bt crops show high efficiency against most target pests, but the risk of insect resistance evolution needs to be given more attention.

## 3. Managing the efficacy of *Bacillus thuringiensis* crops against target pests

The first insect-resistant Bt-transgenic maize was developed in the United States in 1986, but did not enter commercial production until 1996. Subsequently, three Bt-transgenic maize lines were commercialized in the United States, and in 2017, 59.7 million hectares among 14 countries were planted in transgenic maize [10]. GM cotton, commercially grown for more than 20 years, made up 80% of the cotton grown with a planting area of 24.21 million hectares in 2017. Among the 14 countries that grew GM cotton in 2017, the top four producers were India (11.40 million hectares), United States (4.58 million), Pakistan (3.00 million hectares) and China (2.78 million). Bt soya beans have been grown in seven countries since they were introduced in Brazil in 2013. The planting area of Bt aubergine in Bangladesh has reached 2400 ha. Bt sugarcane (expressing Cry1Ab protein) will also be first commercially grown in Brazil in 2018.

When Bt crops were first planted, target pests were effectively controlled, but with the long-term cultivation of Bt crops, target pests gradually developed resistance. To delay the evolution of Bt resistance, refuge strategies are recommended. The success of such strategies depends on three factors: inheritance of the resistance allele must be recessive, resistance allele frequency must be low, and abundant non-Bt host plants must be near the Bt crop [10]. Second-generation Bt crops, that produce two or more distinct Bt toxins, have also been developed and used in target pest resistance management. In some countries, Bt resistance has been delayed with this strategy, while others have failed.

**Figure 1.**
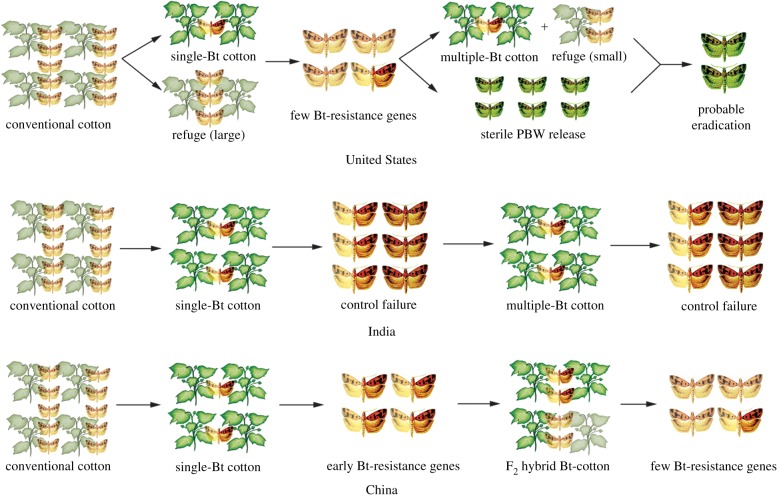
Models of the evolution of pink bollworm (PBW) Bt resistance. In the United States, the effective implementation of high-dose/refuge strategies when growing single-Bt cotton (i.e. a line with one Bt toxin) has maintained PBW populations with few Bt-resistant genes. With planting of multiple-Bt cotton (i.e. a line with more than one Bt toxin), the scale of refuge has been greatly reduced; however, when the line was grown using other control measures, PBW populations were eradicated [11,75]. In India, the cultivation of single-Bt cotton and the lack of refuge have led to Cry1Ac resistance in PBW, resulting in widespread control failures of Bt cotton. Several years after multiple-Bt cotton was planted, PBW sensitivity to multiple-Bt cotton also decreased [30,79]. In China, with the cultivation of Bt cotton, the frequency of PBW resistance increased. After F_2_ Bt cotton was planted, the frequency of resistance decreased because the F_2_ seeds contained 25% conventional cotton as a refuge [82]. Note: light-coloured cotton plants represent conventional cotton, dark-green plants represent Bt cotton, light PBWs represent Bt-sensitive population, dark ones represent Bt-resistant population. PBWs with two colours represent those that carry the resistance mutation genes; green ones represent sterile PWBs.

Pest resistance management can be divided into three types, which we discuss using *P. gossypiella* as an example. In the United States, refuges with non-Bt cotton have grown more than 25% in acreage every year from 1996 to 2005, increasing the survival of the susceptible pink bollworm. Very few resistant pink bollworms in Bt cotton fields mate with the susceptible ones from the refuges because the resistant inheritance is recessive; Bt cotton kills any heterozygous progeny produced by mating between a homozygous susceptible moth and homozygous resistant moth [74,75]. This refuge strategy plays a crucial role in sustaining the susceptibility of the pink bollworm to Bt cotton. With this strategy, even after many years of commercial cultivation of Bt cotton, a few Bt-resistant genes in pink bollworm were detected in fields, and pink bollworm remained susceptible to Bt toxins and was rare in fields [10] (figure 1). With the production of two-toxin cotton, the refuge abundance was greatly reduced during 2006–2009, to a mean percentage of only 7% [75]. Mass releases of sterile pink bollworms in these years has contributed greatly to the control of pink bollworm, and this target pest has nearly been eradicated.

In India, Bt cotton that produces a single Cry1Ac protein has been planted since 2003, and pink bollworms resistant to Bt cotton expressing Cry1Ac were first detected in 2008 in Gujarat [30,76]. This emergence of field-evolved resistance is probably owing to insufficient planting of conventional cotton as refuges [77]. Although the Indian government has mandated that each Cry1Ac cotton field be surrounded by non-Bt refuges with more than five lines or at least 20% of the field area, Indian growers have not complied [78]. Second-generation Bt cotton (expressing Cry1Ac and Cry2Ab protein) has been planted since 2006, and subsequently, one-toxin and two-toxin plants have been grown concurrently [79]. On the basis of continuous field surveys from 2010 to 2017, the survival of pink bollworm on two-toxin Bt cotton increased in central and southern India [80], meaning that the management strategy against Bt resistance in the targeted cotton pest failed in India (figure 1).

In China, millions of small-scale farmers first planted transgenic cotton producing Cry1Ac in 2000 in the Yangtze River Valley to prevent and control pink bollworm [81]. Pest resistance to Cry1Ac toxin increased significantly from 2005–2007 to 2008–2010 in the Yangtze River region. Surprisingly, however, resistance then decreased from 2011 to 2015. After a survey in 2010 and subsequent years, Wan *et al*. [82] found that growers had planted seeds from second-generation (F_2_) cotton hybrids. The production of F_1_ hybrid seeds requires expensive artificial pollination, but F_2_ hybrid seed is relatively easy to produce through self-pollination of plants from F_1_ hybrid seeds at an expected rate of 25% homozygous and 50% heterozygous for Bt toxin production and 25% homozygous for nonproduction of Bt. Thus, the seed mixture generated with F_2_ hybrids is equivalent to the mixture provided by refuges and was the main reason for the delay in Bt resistance of pink bollworm in China (figure 1).

Natural refuges can usually serve as adequate refuges. Owing to intercropping with multiple crops, cotton bollworm has been well controlled and Bt resistance effectively delayed [83]. The effectiveness of natural refuges is influenced by many factors, including the characteristics of target pests, distribution and abundance of host plants, and so on [84]. Although natural refuges are important in delaying Bt resistance in pests, they are not as effective as non-Bt cotton refuges. Field population monitoring data showed that non-recessive resistance increased faster than recessive resistance. During resistance monitoring in 17 counties in six provinces in northern China from 2010 to 2013, Jin *et al*. [85] found that the proportion of resistance among more than 70 000 larvae increased from 1% in 2010 to 5.5% in 2013. This large-scale field investigation and simulation modelling of the evolution of Bt resistance of bollworm in northern China, generated more attention on the increase in non-recessive resistance populations by comparing the developmental trends in non-recessive resistant and recessive resistant populations. Although the overall Bt-resistance levels in the field are still low, the populations of cotton bollworm in China should be monitored carefully for resistance in the future.

In some countries and regions, owing to the widespread planting of Bt-transgenic corn, cotton and other crops, the natural refuge of target pests has disappeared, and the risk of Bt resistance evolution increased dramatically. Fall armyworm (*Spodoptera frugiperda*) is a major maize pest in Brazil, and migrated to South America [86]. Because of wide planting of Bt crops and no natural refuge, this pest had developed resistance to Bt crops [87].

## 4. Resistance mechanisms of target pests to *Bacillus thuringiensis* crops

**Figure 2.**
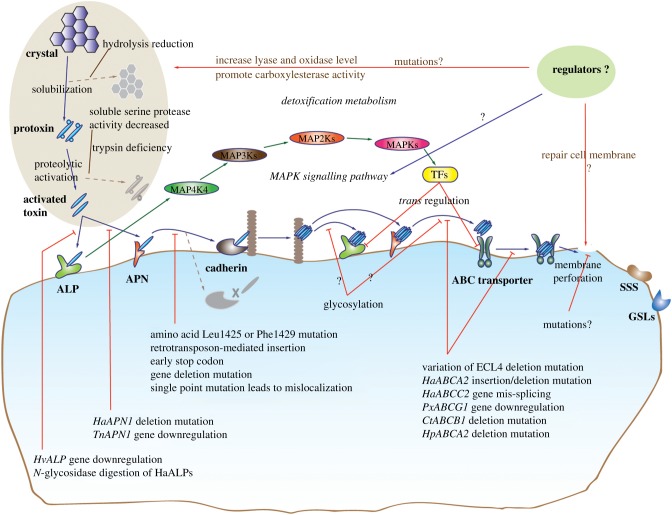
Bt-resistance mechanisms in target pests. The main mechanisms include disruption of the activation of Bt protoxin, mutations or regulation of Bt receptors such as cadherin, ATP binding cassette (ABC) transporters, alkaline phosphatases (ALPs), and aminopeptidase N (APNs), and changes in immune systems. In Bt-resistant insects, activation of Bt toxin and binding of specific receptors on the midgut membrane can activate the mitogen-activated protein kinase (MAPK) signalling pathway, reducing the expression level of Bt-receptor genes via different transcription factors. The MAPK pathway and other regulators may enhance resistance to Bt toxin through the repair of cell membrane damage and changes in the immune system. SSS, sodium solute symporter; GSL, glycosphingolipids; TF, transcription factor.

Laboratory and field data have shown that different mechanisms are involved in the evolution of resistance to Bt crops. So far, the mechanisms comprise three types: variations in toxin activation, mutation in the toxin receptor and regulation of the immune system (figure 2).

### (a) Variations in toxin activation

Bt protoxin is hydrolysed in the alkaline intestine and activated by protease degradation in the midgut, then released as the insecticidal toxin. Changes in the proteases in insect midgut can thus affect the activation of insecticidal proteins. When major intestinal trypsin was absent in the midgut of a Bt-resistant strain of *Plodia interpunctella*, the protoxin was not activated in the midgut, and resulted in Bt resistance [88,89]. Forcada *et al*. [90] reported that changes in the composition of midgut protease in Bt-resistant *H. virescens* strain were associated with a significant reduction in protoxin activation. When the protease activity in resistant and sensitive strains of European corn borer was compared, soluble serine protease activity in sensitive strains was higher than in the resistant strains [91]. Liu *et al*. [92] found that mutations in the promotor of one trypsin gene conferred high Cry1Ac resistance in the cotton bollworm. Although reports have shown that variations in toxin activation are important in the development of Bt resistance [93,94], most researchers believe that the proportion of Bt-resistance cases caused by changes of protease is not very high.

### (b) Mutation in genes for toxin receptors

Midgut membrane-bound cadherin (CAD), ATP binding cassette (ABC) transporters, aminopeptidase N (APN), alkaline phosphatase (ALP) and perhaps unknown receptors have important roles in the insecticidal activity of Bt toxins in lepidopteran larvae. Mutations and gene expression regulation of receptors are important reasons for Bt resistance in insects (table 2).

**Table 2.** Bt-resistance mechanism in target pests.

target pest	receptor/enzyme	resistance mechanism	Bt toxin	reference
*Bombyx mori*	ABCC2	variation in amino acid residues around 770DYWL773 of ECL4	Cry1Aa	[95]
*Chilo suppressalis*	ALP	downregulation	Cry1A Cry2A Cry1C	[96]
*Helicoverpa armigera*	cadherin	premature stop codon	Cry1Ac	[97]
	trypsin	mutations in promoter region	Cry1Ac	[92]
	ABCA2	three independent indel mutations	Cry2Ab	[98]
	ABCC2	insertion of 73 bp in cDNA leads to 6-bp deletion at splicing site	Cry1Ac	[99]
	APN1	deletion mutation	Cry1Ac	[100]
	protease	altered protease profile leads to improper processing of the protoxin	Cry1Ac	[101]
	cadherin	point mutation leads to cadherin mislocalization	Cry1Ac	[102]
	ALP	*N*-glycosidase digestion	Cry1Ac	[103]
*Helicoverpa punctigera*	ABCA2	deletion of 14 bp leads to loss of tpm2 transporter motif in NBF2	Cry2Ab	[98]
*Heliothis virescens*	cadherin	retrotransposon-mediated insertion	Cry1Ac	[104]
	cadherin	single-nucleotide mutation, CTG→CGG	Cry1A	[105]
	ALP	downregulation	Cry1Ac	[106]
	ABCC2	inactivating mutation	Cry1Ac	[107]
*Ostrinia furnacalis*	ABCG1	knockdown	Cry1Ab Cry1Ac	[108]
	cadherin	downregulation and mutation	Cry1Ac	[109]
*Ostrinia nubilalis*	cadherin	premature termination codons and/or large deletions	Cry1Ab	[110]
	ABCC2	mutation	Cry1Fa	[111]
	APN	downregulation	Cry1Ab	[112]
	Aminopeptidase-P like gene	mutation	Cry1Ab	[113]
*Pectinophora gossypiella*	cadherin	three mutant alleles in toxin-binding region	Cry1Ac	[114]
	cadherin	deletion of 207 bp and loss of transmembrane domain	Cry1Ac	[115]
	cadherin	premature stop codon, deletion of at least 99 bp or both	Cry1Ac	[116]
	cadherin	insertion of intact CR1 retrotransposon	Cry1Ac	[117]
*Plutella xylostella*	ABCG1	downregulation mediated by MAPK pathway	Cry1Ac	[118]
	ALP	downregulation mediated by MAPK pathway	Cry1Ac	[118]
	ABCC2	mutation	Cry1Ac	[119]
*Spodoptera exigua*	ALP2	knockdown	Cry2Aa	[120]
	APN	downregulation	Cry1Ca	[121]
	ABCC2	mutation	Cry1Ac Cry1Ca	[122]
*Spodoptera frugiperda*	ALP	downregulation	Cry1Fa	[123]
*Trichoplusia ni*	APN1	downregulation	Cry1Ac	[124]

#### (i) Cadherin

CAD is one of the most important Bt toxin receptors because it has important roles in toxin oligomerization. Bt resistance in many pests is related to a mutation in the *CAD* gene. Disruption of the gene by retrotransposon-mediated insertion and an early stop codon is related to the high resistance to Cry1Ac toxin that developed in the cotton pest *H. virescens* [104]. Morin *et al*. [114] reported that pink bollworm field populations harboured three mutant alleles of the *CAD*-encoding gene that were linked to Cry1Ac resistance. Each of the three Cry1Ac-resistance alleles had a deletion, which was associated with binding of Cry1Ac. Zhao *et al*. [125] also reported that diverse CAD mutations in cotton bollworm were linked with resistance to Cry1Ac toxin. In addition, a CAD transmembrane mutation affects cellular trafficking and results in resistance of pink bollworm to Cry1Ac toxin [115]. Amino acids Leu^1425^ and Phe^1429^ play a vital role in the interaction between CAD and Cry1Ac toxin, and if they are replaced with charged amino acids, the toxin will not bind to CAD, which may lead to resistance to Cry1Ac [106]. Xiao *et al*. [102] found that a single-point mutation caused CAD mislocalization on the surface of the midgut epithelium, which led to high Cry1Ac resistance in the cotton bollworm, which is a novel finding. The interaction of CAD1 and CAD2 with Bt toxins may underlie Bt resistance in the important rice pest *C. suppressalis*; reducing the expression of CAD1 or CAD2 can increase resistance to Cry2A and Cry1C [108].

#### (ii) ATP binding cassette transporter

ABC transporter plays important roles in the toxicity of Bt toxin and insect metabolism of chemical pesticides. A mutation in *ABCC2* was first found to contribute to Cry1Ac resistance in *H. virescens* [107], then in other insects such as *Bombyx mori* and *Plutella xylostella* [126,127]. Xiao *et al*. [99] demonstrated that mis-splicing of the *ABCC2* gene led to a loss of 150 amino acids and conferred high resistance to Cry1Ac toxin in *H. armigera*. Tay *et al*. [98] found that a mutation in the *ABCA2* gene in cotton bollworm led to resistance to Cry2Ab toxin, another important Bt toxin used in cotton. This finding was the first elucidation of a molecular genetic mechanism resistance to Cry2Ab in insects, and the detection of related resistance sites was helpful to understand the microevolution processes of Bt resistance in lepidopteran insects. Wang *et al*. [128] knocked out the midgut *HaABCA2* gene with the clustered regularly interspaced short palindromic repeats (CRISPR)/Cas9 gene editing system and revealed that the edited strains had high levels of resistance to Cry2Aa and Cry2Ab. The ABCG1 protein is located on the cell membrane, and expression of the *ABCG1* gene in a Bt-resistant population of *P. xylostella* population is significantly lower than in the susceptible populations. Silencing by RNA interference (RNAi) of the midgut *ABCG1* gene significantly reduces susceptibility of *P. xylostella* to Cry1Ac toxin. Moreover, decreased expression of the *ABCG1* gene is closely linked to resistance to Cry1Ac [129]. Downregulation of genes in the *ABCG* subfamily of *Ostrinia furnacalis* is related to its resistance to Cry1Ab and Cry1Ac [12]. The mitogen-activated protein kinase (MAPK) signalling pathway alters expression of *ABCC* genes, leading to high resistance to Cry1Ac in *P. xylostella* [118]. Recently, forkhead box protein A (FOXA) was also shown to upregulate expression of *ABCC2* and *ABCC3* genes in *S. frugiperda* SF9 cells [126]. ABCC2 and ABCC3 are important receptors of Cry1Ac toxin, so the low expression of FOXA is related to the lepidopteran larval resistance to Bt toxin. Specific toxicity of Cry1Aa to some lepidopteran insects is related to the conservation or variation in amino acid residues around ^770^DYWL^773^ of extracellular loop 4 (ECL4) in ABCC2 [95]. ABC transporters may play key roles together with CAD, which is responsible for oligomerization of activated toxins and may be necessary for binding to ABC transporters. ABC transporters might bind to the oligomeric toxins to form pores. Perhaps in some insects, the oligomerization is not needed; thus, CAD would not function in the insecticidal process in these insects. But ABC transporters are necessary. Different ABC transporters can bind to different Bt toxins, for example, ABCA2 is the receptor for Cry2Ab, and ABCC2 is the receptor for Cry1Ab and Cry1Ac [98]. We speculate that nearly all Bt toxins need a responding ABC transporter to bind and forming pores. Moreover, a strain with a mutation in the *ABCC2* gene is more sensitive to abamectin, which means that Bt resistance which is mediated by the ABC transporter mutation may cause negative cross-resistance to other biological or chemical insecticides [127].

#### (iii) Alkaline phosphatase

In the brush border membrane vesicle of *H. virescens*, ALP is a receptor for Cry1Ac toxin [106], and ALP levels in resistant *H. virescens* are significantly lower than in susceptible strains. According to proteomic and genomic analyses of the Bt-resistant and susceptible larvae of *H. virescens*, *H. armigera* and *S. frugiperda*, the level of ALP that bound to the midgut membrane is significantly lower in resistant strains than in susceptible [130]. The MAPK signalling pathway alters expression of *ALP* genes, causing Cry1Ac resistance in *P. xylostella* [118]. When the *ALP* gene is downregulated, *C. suppressalis* becomes resistant to Cry1A-, Cry2A- and Cry1C-transgenic rice lines [96]. ALP2 is also important for the susceptibility of *Spodoptera exigua* to Cry2Aa and is probably the receptor for Cry2Aa [120]. The expression of midgut membrane-bound Cry1Fa and midgut ALP is also reduced in a field-evolved Bt-resistant *S. frugiperda* strain [123]. In an analysis of the molecular mechanism of HaALP binding to Cry1Ac toxin in *H. armigera*, Ning *et al*. [103] found that *N*-glycosidase digestion of HaALPs reduces the binding level of Cry1Ac on the midgut brush border membrane surface. The exact function of ALPs as important receptors for Bt toxins is still unclear. One of our hypotheses is that the glycosyl on ALP binds the toxins, which may help the toxin accumulate, accelerate oligomerization of the Bt toxin by CAD and eventually cause cell perforation by binding to the ABC transporters.

#### (iv) Aminopeptidase N

APN is also an important receptor in the midgut membrane of insects for Bt toxins. Zhang *et al*. [100] reported that HaAPN1 was a receptor of Cry1Ac, and a deletion mutation in the HaAPN1 gene is associated with resistance of *H. armigera* to Cry1Ac. When the *HaAPN1* gene is silenced by RNAi, the susceptibility of *H. armigera* to Cry1Ac is reduced [131]. Biochemical, proteomic, and molecular analyses of Cry1Ac-resistant cabbage loopers revealed that *APN1* gene expression is significantly downregulated, but *ANP6* gene expression is significantly upregulated. Further analysis showed that Cry1Ac resistance is only related to the downregulation of APN1. The concurrent upregulation of *APN6* might play a compensating role for the loss of APN1 to minimize the fitness costs of resistance [124]. In a comparison of Cry1Ab-resistant and -susceptible strains of *O. furnacalis*, the APN sequence of the resistant strain had an amino acid variation in four locations [132]. An RNAi-mediated knockdown analysis showed that APN1, APN3 and APN6 might be receptors of Cry1Ca in *S. exigua* [133]. However, the role of APNs in the Bt-insecticidal process is not very clear. Perhaps their role is similar to that of ALPs.

#### (v) Other receptors

Other types of receptors on the cell membrane are involved in insect interactions with Bt toxin. For example, several possible Cry3Ba receptors in *Tribolium castaneum* were identified by ligand blotting. Sodium solute symporter (TcSSS) protein gene knockdown enhances the resistance of *T. castaneum* to Cry3Ba. The presence of CAD repeats in amino acid sequences is a significant feature of TcSSS, and a TcSSS peptide fragment that contains sequences homologous to binding epitopes in Bt CAD functional receptors was found to enhance Cry3Ba toxicity in *Manduca sexta* and *Tenebrio molitor* [134]. This finding was the first report that the TcSSS protein is a Bt toxin receptor, which broadens the scope of Bt-resistance mechanisms in insects. Bt toxin can bind to glycolipids directly, and Griffitts *et al*. [135] found that Cry1Ac, Cry1Aa and Cry1Ab combine with the same glycolipids extracted from midguts of *M. sexta*. Resistance to Cry1Ac in a strain of *P. xylostella* is also associated with a decrease in glycolipid levels, consistent with glycolipids serving as general host cell receptors for these toxins [136]. Chen *et al*. [137] reported that glucosinolate sulfatases GSS1 and GSS2 bind directly to Cry1Bd in *P. xylostella* and play a crucial role in Cry1Bd toxicity. New Bt receptors and new mechanisms are likely to be discovered as research continues.

### (c) Changes in immune systems

Insects can improve their resistance to Bt toxin by increasing the level of esterses such carboxylesterase or accelerating degradation of the toxin [138,139]. Carboxylic cholinesterase increases in larvae of *M. sexta* after they feed on Bt toxin [90]. In the third-instar larvae of the Asian corn borer, carboxylesterase activity is significantly lower after the larvae feed on Bt maize than on non-Bt maize, indicating that the activity of carboxylesterase may be related to the detoxification of Bt by the insect [139]. In an Australian bollworm population with 275-fold higher resistance to Bt toxin than in the susceptible strain, inheritance of this resistance was found to be autosomal semi-dominant and associated with elevated esterase levels [140]. Biochemical analysis showed that the esterase in the resistant population binds to the Bt protoxin and the activated protoxin, preventing the toxin from binding to the receptor.

Symbiotic microbes in insects may also be involved in insect interactions with Bt toxins. Larvae of *H. armigera* carrying HaDNV-1, a novel densovirus that phylogenetically groups with members of the genus *Iteravirus*, are significantly more resistant to Bt toxin at low doses [141]. Compared with uninfected insects, HaDNV-1-positive individuals develop faster and have greater reproductive capacity. These results suggest that HaDNV improves the resistance of *H. armigera* to Bt cotton and helps the pest survive in Bt crop areas. Perhaps the interaction between an insect pest and a microorganism can activate immunity or tolerance in the pest, increase its rate of growth and reduce the fitness cost for Bt resistance. In the laboratory, although symbionts seem to contribute little to Bt-resistance levels in pests, in the natural environment, the effect might be remarkable.

## 5. Discussion

With further research and commercialization of multiple-gene Bt crops, the efficacy of pest control can be improved and the development of Bt resistance delayed. Usually, Bt genes have different insecticidal mechanisms, thus providing choices for a particular Bt crop. When the target pest evolves resistance to one Bt toxin, another Bt toxin still can kill them. Moreover, the percentage refuge can be greatly reduced when multiple-Bt-gene crops are planted. In the United States and Australia, the non-Bt cotton refuge was more than 25% of the area planted to cotton with a single-Bt toxin protein. For cotton expressing two Bt proteins, the area of the refuge has been decreasing significantly [74,75]. This reduction is even more important for some developing countries such as China and India, where there are many small farmers, and they do not want to plant conventional crops as the refuge.

The discovery of new Bt genes is another important direction for Bt crop development, primarily in two areas: new Bt genes with different insecticidal mechanisms that can kill the target pests that are now resistant to previous Bt toxins and Bt genes to control important hemipteran pests such as mirids, planthopper, aphids rather than lepidopteran and coleopteran insects. Towards this new direction, Monsanto has developed GM cotton MON88702, which produces a modified Cry51Aa2 toxin protein with good insecticidal activity against hemipteran insects [16]. Another modified Bt-Cyt2Aa crystal toxin is toxic to green peach aphids and pea aphids [142]. So crops with Bt genes that control a wider range of pests seem likely in the future.

Another important area is increasing the commercialization of Bt crops. Although Bt maize has been studied for many years and is very successful in controlling pests and reducing usage of insecticides, Bt maize is still not commercialized in China. People worry about the food safety of Bt maize [10]. Relevant policies should be further enriched to drive the use of Bt maize. Bt rice and other Bt crops encounter the same problems. Other countries face similar problems and worries.

There are legitimate concerns with commercializing more Bt that still need to be addressed. Pest populations, especially those of polyphagous insects, may be affected by the commercialization of many crops. Usually, Bt resistance in polyphagous insects can be delayed by mass migrations in different areas and different crops. Conventional crops in different areas can serve as natural refuges [83]. If the non-Bt crops are replaced by Bt crops, the insects will continue to be under high Bt selection pressure, and the evolution of Bt resistance will be accelerated, which will increase the difficulty of pest control. In Brazil, with the large-scale planting of Bt maize and Bt cotton, the rate of Bt-resistance emergence among fall armyworms (*S. frugiperda*) has increased dramatically. Most Bt maize varieties gradually lost their ability to control fall armyworm after only 3 years of planting [87]. Fall armyworms can also migrate long distances. In 2016 for the first time, this pest was found in South Africa and caused significant damage to maize crops and other crops [143]. It has spread to almost all of Africa [144].

Integrated pest management that combines the use of attractants; physical, chemical and biological controls; and planting both Bt and non-Bt crops helps delay the evolution of insect resistance. In recent years, new molecular techniques have been applied to pest control to indirectly help to reduce the harm from resistant pests. Host-mediated RNAi of important pest genes has been proposed as a potential avenue for increasing crop resistance against pests. Plants that have been modified to express double-stranded RNA against suitable target genes in pests have effectively controlled pest growth and reproduction or reduced pest resistance to pesticides [145,146]. Ni *et al*. [147] demonstrated by computer simulation that, compared with Bt cotton alone, Bt cotton combined with RNAi can substantially delay the evolution of Bt resistance in bollworm. CRISPR/Cas9-mediated knockout of related genes effectively inhibits egg production and viability of target pests [148]. CRISPR/Cas9 technology provides an easier way to control pests using a site-specific homing-based gene driver, as demonstrated in model insects [149]. If CRISPR-based gene drivers can be used to spread target genetic elements through wild populations and then combined with Bt-transgenic crops, we may have a more effective measure for pest resistance management. In addition, as mentioned earlier, a resistant bollworm strain with a mutation in *ABCC2* had negative cross-resistance between Cry1Ac and abamectin [127]; thus, taking advantage of the fitness cost of resistance may provide another strategy for managing resistance. If similar negative cross-resistance mechanisms exist in other pests, new methods will be developed to prevent and control the pests [150]. Such research provides a theoretical basis for feasible strategies to manage Bt resistance and support the long-term usage of multiple Bt crops. With the development of genomics, proteomics and metabolomics, we anticipate more novel integrated pest control technologies will be developed and adopted. Such technologies and other environment-friendly pest control methods will provide safer, more effective pest management.

## Data accessibility

This article has no additional data.

## References

[1] Sparks TC, Nauen R 2015 IRAC: mode of action classification and insecticide resistance management. Pestic. Biochem. Phys. 121, 122–128. (10.1016/j.pestbp.2014.11.014) 26047120

[2] Davies TGE, Field LM, Williamson MS 2012 The re-emergence of the bed bug as a nuisance pest: implications of resistance to the pyrethroid insecticides. Med. Vet. Entomol. 26, 241–254. (10.1111/j.1365-2915.2011.01006.x) 22235873

[3] Hanazato T 2001 Pesticide effects on freshwater zooplankton: an ecological perspective. Environ. Pollut. 112, 1 (10.1016/S0269-7491(00)00110-X) 11202648

[4] Liu Q, Hallerman E, Peng Y, Li Y 2016 Development of Bt rice and Bt maize in China and their efficacy in target pest control. Int. J. Mol. Sci. 17, E1561 (10.3390/ijms17101561) 27763554 PMC5085622

[5] Dai WB 2013 Research on prevention and control of Chinese agricultural ecological environment pollution to ensure food safety. Adv. Mater. Res. 616–618, 2247–2250. (10.4028/www.scientific.net/AMR.616-618.2247)

[6] Bravo A, Gill SS, Soberón M 2007 Mode of action of *Bacillus thuringiensis* Cry and Cyt toxins and their potential for insect control. Toxicon 49, 423–435. (10.1016/j.toxicon.2006.11.022) 17198720 PMC1857359

[7] Gill SS, Cowles EA, Pietrantonio PV 1992 The mode of action of *Bacillus thuringiensis* endotoxins. Annu. Rev. Entomol. 37, 615 (10.1146/annurev.en.37.010192.003151) 1311541

[8] Bravo A, Likitvivatanavong S, Gill SS, Soberón M 2011 *Bacillus thuringiensis*: a story of a successful bioinsecticide. Insect. Biochem. Mol. Biol. 41, 423–431. (10.1016/j.ibmb.2011.02.006) 21376122 PMC3689885

[9] Betz FS, Hammond BG, Fuchs RL 2000 Safety and advantages of *Bacillus thuringiensis*-protected plants to control insect pests. Regul. Toxicol. Pharm. 32, 156 (10.1006/rtph.2000.1426) 11067772

[10] ISAAA. 2017 Global status of commercialized biotech/GM crops in 2017: biotech crop adoption surges as economic benefits accumulate in 22 years. *ISAAA Brief* No. **53**. ISAAA, Ithaca, NY See http://www.isaaa.org/purchasepublications/itemdescription.asp?ItemType=BRIEFS&Control=IB053-2017.

[11] Tabashnik BE, Brévault T, Carrière Y 2013 Insect resistance to Bt crops: lessons from the first billion acres. Nat. Biotechnol. 31, 510–521.(10.1038/nbt.2597) 23752438

[12] Zhang TT, Coates BS, Wang YQ, Wang YD, Bai SX, Wang ZY, He KL 2017 Down-regulation of aminopeptidase N and ABC transporter subfamily G transcripts in Cry1Ab and Cry1Ac resistant Asian corn borer, *Ostrinia furnacalis* (Lepidoptera: Crambidae). Int. J. Biol. Sci. 13, 835–851. (10.7150/ijbs.18868) 28808417 PMC5555102

[13] Crava CM, Yolanda B, Jakubowska AK, Juan F, Baltasar E 2013 Midgut aminopeptidase N isoforms from *Ostrinia nubilalis*: activity characterization and differential binding to Cry1Ab and Cry1Fa proteins from *Bacillus thuringiensis*. Insect. Biochem. Mol. Biol. 43, 924–935. (10.1016/j.ibmb.2013.07.009) 23933214

[14] Ríosdíez JD, Siegfried B, Saldamandobenjumea CI 2017 Susceptibility of *Spodoptera frugiperda* (Lepidoptera: Noctuidae) strains from central Colombia to Cry1Ab and Cry1Ac entotoxins of *Bacillus thuringiensis*. Southwest Entomol. 37, 281–293. (10.3958/059.037.0304)

[15] George DM, Rind FC, Bendall MW, Taylor MA, Gatehouse AM 2012 Developmental studies of transgenic maize expressing Cry1Ab on the African stem borer, *Busseola fusca*; effects on midgut cellular structure. Pest Manag. Sci. 68, 330–339. (10.1002/ps.2260) 21842526

[16] Huang F, Ghimire MN, Leonard BR, Daves C, Levy R, Baldwin J 2012 Extended monitoring of resistance to *Bacillus thuringiensis* Cry1Ab maize in *Diatraea saccharalis* (Lepidoptera: Crambidae). Gm Crops 3, 245–254. (10.4161/gmcr.20539) 22688686

[17] Trisyono YA, Chippendale GM 2002 Susceptibility of field-collected populations of the southwestern corn borer, *Diatraea grandiosella*, to *Bacillus thuringiensis*. Pest Manag. Sci. 58, 1022–1028. (10.1002/ps.551) 12400441

[18] Sharma P, Nain V, Lakhanpaul S, Kumar PA 2010 Synergistic activity between *Bacillus thuringiensis* Cry1Ab and Cry1Ac toxins against maize stem borer (*Chilo partellus swinhoe*). Lett. Appl. Microbiol. 51, 42–47. (10.1111/j.1472-765X.2010.02856.x) 20536706

[19] Santos-Amaya OF, Tavares CS, Rodrigues JVC, Campos SO, Guedes RNC, Alves AP, Pereira EJG 2017 Fitness costs and stability of Cry1Fa resistance in Brazilian populations of *Spodoptera frugiperda*. Pest Manag. Sci. 73, 35–43. (10.1002/ps.4312) 27147125

[20] Siebert MW et al. 2012 Evaluation of corn hybrids expressing Cry1F, Cry1A.105, Cry2Ab2, Cry34Ab1/Cry35Ab1, and Cry3Bb1 against southern United States insect pests. J. Econ. Entomol. 105, 1825–1834. (10.1603/EC12155) 23156183

[21] Gaspers C, Siegfried BD, Spencer T, Alves AP, Storer NP, Schuphan I, Eber S 2011 Susceptibility of European and north American populations of the European corn borer to the Cry1f insecticidal protein. J. Appl. Entomol. 135, 7–16. (10.1111/j.1439-0418.2010.01541.x)

[22] Jansens S et al. 1997 Transgenic corn expressing a Cry9C insecticidal protein from *Bacillus thuringiensis* protected from European corn borer damage. Crop Sci. 37, 1616–1624. (10.2135/cropsci1997.0011183X003700050035x)

[23] Bernardi O, Bernardi D, Ribeiro RS, Okuma DM, Salmeron E, Fatoretto J, Medeiros FCL, Burd T, Omoto C 2015 Frequency of resistance to Vip3Aa20 toxin from *Bacillus thuringiensis* in *Spodoptera frugiperda* (Lepidoptera: Noctuidae) populations in Brazil. Crop Prot. 76, 7–14. (10.1016/j.cropro.2015.06.006)

[24] Kang LS, Wang YM, Jiang Y, Li CC, Xing ZJ, Zhang M 2009 Resistance to maize borer (*Ostrinia furnacalis*) of transgenic Bt maize and yield analysis. J. Maize Sci. 17, 62–64&70.

[25] Frank DL et al 2015 Effect of seed blends and soil-insecticide on western and northern corn rootworm emergence from mCry3A+eCry3.1Ab Bt maize. J. Econ. Entomol. 108, 1260–1270. (10.1093/jee/tov081) 26470254

[26] Zhang YW, Liu YJ, Ren Y, Liu Y, Liang GM, Song FP, Bai SX, Wang JH, Wang GY 2013 Overexpression of a novel Cry1Ie, gene confers resistance to Cry1Ac-resistant cotton bollworm in transgenic lines of maize. Plant Cell Tissue Org. 115, 151–158. (10.1007/s11240-013-0348-5)

[27] Du DX, Geng CJ, Zhang XB, Zhang ZX, Zheng YL, Zhang FD, Lin YJ, Qiu FZ 2014 Transgenic maize lines expressing a cry1C*, gene are resistant to insect pests. Plant Mol. Biol. Rep. 32, 549–557. (10.1007/s11105-013-0663-3)

[28] Kumar GVS, Swamy SVSG 2014 A duo-decennium of Bt cotton adoption in India: an overview. Curr. Biotica. 8, 322–340.

[29] Lee MK, Miles P, Chen JS 2006 Brush border membrane binding properties of *Bacillus thuringiensis* Vip3A toxin to *Heliothis virescens* and *Helicoverpa zea* midguts. Biochem. Bioph. Res. Co. 339, 1043–1047. (10.1016/j.bbrc.2005.11.112) 16337146

[30] Dhurua S, Gujar GT 2011 Field-evolved resistance to Bt toxin Cry1Ac in the pink bollworm, *Pectinophora gossypiella* (Saunders) (Lepidoptera: Gelechiidae), from India. Pest Manag. Sci. 67, 898–903. (10.1002/ps.2127) 21438121

[31] Downes S, Parker TL, Mahon RJ 2009 Frequency of alleles conferring resistance to the *Bacillus thuringiensis* toxins Cry1Ac and Cry2Ab in Australian populations of *Helicoverpa punctigera* (Lepidoptera: Noctuidae) from 2002 to 2006. J. Econ. Entomol. 102, 733–742. (10.1603/029.102.0234) 19449655

[32] Qiu L et al. 2015 Cadherin is involved in the action of *Bacillus thuringiensis*, toxins Cry1Ac and Cry2Aa in the beet armyworm, *Spodoptera exigua*. J. Invertebr. Pathol. 127, 47–53. (10.1016/j.jip.2015.02.009) 25754522

[33] Li YX, Greenberg SM, Liu TX 2006 Effects of Bt cotton expressing Cry1Ac and Cry2Ab and non-Bt cotton on behavior, survival and development of *Trichoplusia ni*, (Lepidoptera: Noctuidae). Crop Prot. 25, 940–948. (10.1016/j.cropro.2005.12.007) 17421053

[34] Sivasupramaniam S et al. 2008 Toxicity and characterization of cotton expressing *Bacillus thuringiensis* Cry1Ac and Cry2Ab2 proteins for control of lepidopteran pests. J. Econ. Entomol. 101, 546–554. (10.1603/0022-0493(2008)101[546:TACOCE]2.0.CO;2) 18459423

[35] Walsh TK, Downes SJ, Gascoyne J, James W, Parker T, Armstrong J, Mahon RJ 2014 Dual Cry2Ab and Vip3A resistant strains of *Helicoverpa armigera* and *Helicoverpa punctigera* (Lepidoptera: Noctuidae); testing linkage between loci and monitoring of allele frequencies. J. Econ. Entomol. 107, 1610–1617. (10.1603/EC13558) 25195454

[36] Carrière Y, Degain BA, Unnithan GC, Harpold G, Heuberger S, Li XC, Tabashnik BE 2017 Effects of seasonal changes in cotton plants on evolution of resistance to pyramided Cry1Ac +Cry1F cotton by *Helicoverpa zea*. Pest Manag. Sci. 74, 627–637. (10.1002/ps.4746) 28967711

[37] Dennehy TJ 2014 Baseline susceptibility of bollworm (*Helicoverpa zea*) and tobacco budworm (*Heliothis virescens*) larvae to Cry1Ab and Cry2Ae Bt proteins. Conference report. See https://esa.confex.com/esa/2015seb/webprogram/Paper90525.html.

[38] Kim HS, Noh S, Park Y 2017 Enhancement of *Bacillus thuringiensis*, Cry1Ac and Cry1Ca toxicity against *Spodoptera exigua*, (Hübner) by suppression of a chitin synthase b gene in midgut. J. Asia-Pacific Entomol. 20, 199–205. (10.1016/j.aspen.2016.12.015)

[39] Baum JA et al. 2012 Cotton plants expressing a hemipteran-active *Bacillus thuringiensis* crystal protein impact the development and survival of *Lygus hesperus* (Hemiptera: Miridae) nymphs. J. Econ. Entomol. 105, 616–624. (10.1603/EC11207) 22606834

[40] Zhang XW, Shu CL, Lu YH, Liu CY, Zhang J, Gao JG 2016 Screening of Bt isolates with insecticidal activity against *Apolygus lucorum* (Heteroptera: Miridae) and bioassay of Cry15Aa polypeptides. Plant Prot. 42, 56–62.

[41] Xu XL, Wang FS, Liu ZR, Yao YJ, Ji XY, Jiang JX 2017 Impacts of transgenic Cry1Ab / Cry1Ac rice ganlü 1 on *Cnaphalocrocis medinalis* guenée and *Chilo suppressalis* (Walker) and their arthropod predators under field conditions. Acta Phytophyl. Sin. 44, 1–7.

[42] Tu JM, Zhang GA, Datta K, Xu CG, He YQ, Zhang QF, Khush GS, Datta SK 2000 Field performance of transgenic elite commercial hybrid rice expressing *Bacillus thuringiensis* δ-endotoxin. Nat. Biotechnol. 18, 1101–1104. (10.1038/80310) 11017051

[43] Tang H, Chen G, Chen F, Han L, Peng Y 2018 Development and relative fitness of Cry1C resistance in *Chilo suppressalis*. Pest Manag. Sci. 74, 590–597. (10.1002/ps.4740) 28941326

[44] Zheng X, Yang Y, Xu H, Chen H, Wang B, Lin Y, Lu Z 2011 Resistance performances of transgenic Bt rice lines T2a-1 and T1c-19 against *Cnaphalocrocis medinalis* (Lepidoptera: Pyralidae). J. Econ. Entomol. 104, 1730–1735. (10.1603/EC10389) 22066204

[45] Chen H, Zhang G, Zhang Q, Lin Y 2008 Effect of transgenic *Bacillus thuringiensis* rice lines on mortality and feeding behavior of rice stem borers (Lepidoptera: Crambidae). J. Econ. Entomol. 101, 182–189. (10.1093/jee/101.1.182) 18330134

[46] Chen H, Tang W, Xu C, Li X, Lin Y, Zhang Q 2005 Transgenic indica rice plants harboring a synthetic Cry2a* gene of *Bacillus thuringiensis* exhibit enhanced resistance against lepidopteran rice pests. Theor. Appl. Genet. 111, 1330–1337. (10.1007/s00122-005-0062-8) 16187120

[47] Chen Y, Tian JC, Shen ZC, Peng YF, Hu C, Guo YY, Ye GY 2010 Transgenic rice plants expressing a fused protein of Cry1Ab /Vip3 h has resistance to rice stem borers under laboratory and field conditions. J. Econ. Entomol. 103, 1444–1453. (10.1603/EC10014) 20857760

[48] Liu YL, Wang YL, Shu CL, Lin KJ, Song FP, Bravo A, Soberón M, Zhang J 2018 Cry64Ba and Cry64Ca, two ETX/MTX2-type *Bacillus thuringiensis* insecticidal proteins active against hemipteran pests. Appl. Environ. Microbiol. 84, e01996-17 (10.1128/AEM.01996-17) 29150505 PMC5772221

[49] Zhou ZX, Pang JH, Guo WC, Zhong NQ, Tian YC, Xia GX, Wu JH 2012 Evaluation of the resistance of transgenic potato plants expressing various levels of Cry3a against the Colorado potato beetle (*Leptinotarsa decemlineata* say) in the laboratory and field. Pest Manag. Sci. 68, 1595–1604. (10.1002/ps.3356) 22807197

[50] Kumar M, Chimote V, Singh R, Mishra GP, Naik PS, Pandey SK, Chakrabarti SK 2010 Development of Bt transgenic potatoes for effective control of potato tuber moth by using Cry1Ab gene regulated by gbss promoter. Crop Prot. 29, 121–127. (10.1016/j.cropro.2009.11.001)

[51] Yu HL, Li YH, Li XJ, Romeis J, Wu KM 2013 Expression of Cry1Ac in transgenic Bt soybean lines and their efficiency in controlling lepidopteran pests. Pest Manag. Sci. 69, 1326–1333. (10.1002/ps.3508) 23564718

[52] Walker DR, All JN, Mcpherson RM, Rogerboerma H, Parrott WA 2000 Field evaluation of soybean engineered with a synthetic Cry1Ac transgene for resistance to corn earworm, soybean looper, *Velvetbean caterpillar* (Lepidoptera: Noctuidae), and lesser cornstalk borer (Lepidoptera: Pyralidae). J. Econ. Entomol. 93, 613–622. (10.1603/0022-0493-93.3.613) 10902306

[53] Ranjithkumar L, Patil BV, Ghante VN, Bheemanna M, Arunkumar H 2013 Baseline sensitivity of brinjal shoot and fruit borer, *Leucinodes orbonalis* (guenée) in south India to Cry1Ac insecticidal protein of *Bacillus thuringiensis*. Curr. Sci. 105, 366–370.

[54] Wu X, Huang F, Leonard BR, Moore SH 2007 Evaluation of transgenic *Bacillus thuringiensis* corn hybrids against Cry1Ab-susceptible and -resistant sugarcane borer (Lepidoptera: Crambidae). J. Econ. Entomol. 100, 1880–1886. (10.1603/0022-0493) 18232406

[55] Huang F, Buschman LL, Higgins RA, Mcgaughey WH 1999 Inheritance of resistance to *Bacillus thuringiensis* toxin (dipel es) in the European corn borer. Science 284**,** 965–967. (10.1126/science.284.5416.965) 10320377

[56] Masson L, Schwab G, Mazza A, Brousseau R, Potvin L, Schwartz JL 2004 A novel *Bacillus thuringiensis* (PS149B1) containing a Cry34Ab1/Cry35Ab1 binary toxin specific for the western corn rootworm *Diabrotica virgifera virgifera* leconte forms ion channels in lipid membranes. Biochemistry 43, 12 349–12 357. (10.1021/bi048946z) 15379574

[57] Johnson KD, Campbell LA, Lepping MD, Rule DM 2017 Field trial performance of herculex XTRA (Cry34Ab1/Cry35Ab1) and SmartStax (Cry34Ab1/Cry35Ab1+Cry3Bb1) hybrids and soil insecticides against Western and Northern corn rootworms (Coleoptera: Chrysomelidae). J. Econ. Entomol. 110, 1062–1069. (10.1093/jee/tox099) 28430986 PMC5444676

[58] Hari NS, Jindal J, Malhi NS 2007 Resistance of Cry1Ab maize to spotted stemborer *Chilo partellus* (Lepidoptera: Crambidae) in India. Int. J. Trop. Insect Sci. 27, 223–228. (10.1017/S1742758407850983)

[59] Meissle M, Romeis J 2009 Insecticidal activity of Cry3Bb1 expressed in Bt maize on larvae of the colorado potato beetle, *Leptinotarsa decemlineata*. Entomol. Exp. Appl. 131, 308–319. (10.1111/j.1570-7458.2009.00859.x)

[60] Shrestha RB, Jakka SRK, Gassmann AJ 2018 Response of Cry3Bb1-resistant western corn rootworm (Coleoptera: Chrysomelidae) to Bt maize and soil insecticide. J. Appl. Entomol. 7, 1–10. (10.1111/jen.12505)

[61] Callestorrez V, Knodel JJ, Boetel MA, Doetkott CD, Podliska KK, Ransom JK, Beauzay P, French BW, Fuller BW 2018 Transgenic Bt corn, soil insecticide, and insecticidal seed treatment effects on corn rootworm (Coleoptera: Chrysomelidae) beetle emergence, larval feeding injury, and corn yield in North Dakota. J. Econ. Entomol. 111, 348–360. (10.1093/jee/tox297) 29186516

[62] Huang Q, Mao L, Huang W, Guo S 1998 Transgenic tobacco plants with a fully synthesized GFM, CryiA gene provide effective tobacco bollworm (*Heliothis armigera*) control. Acta Bot. Sin. 40, 228–233.

[63] Wu KM, Guo YY 2005 The evolution of cotton pest management practices in China. Annu. Rev. Entomol. 50, 31–52. (10.1146/annurev.ento.50.071803.130349) 15355239

[64] Cui JJ, Luo JY, Wang CY, Yan MA, Chun-Hua LI 2004 Population dynamics of main pests and enemies in the transgenic Cry1Ac+CpTi cotton field. Acta Gossypii Sinica. 16, 94–101. (10.3969/j.issn.1002-7807.2004.02.006)

[65] Lu Y, Wu K, Jiang Y, Xia B, Li P, Feng H, Wyckhuys KA, Guo Y 2010 Mirid bug outbreaks in multiple crops correlated with wide-scale adoption of Bt cotton in China. Science 328, 1151–1154. (10.1126/science.1187881) 20466880

[66] Anilkumar G et al. 2016 A transgenic approach for controlling lygus in cotton. Nat. Commun. 7, 12213 (10.1038/ncomms12213) 27426014 PMC4960306

[67] Bortolotto OC, Bueno AF, Braga K, Barbosa GC, Sanzovo A 2014 Biological characteristics of *Heliothis virescens* fed with Bt-soybean MON 87701×MON 89788 and its conventional isoline. An Acad. Bras. Cienc. 86, 973–980. (10.1590/0001-3765201420130495) 30514032

[68] Azambuja R, Degrande PE, Santos ROD, Souza EPD, Gomes CEC 2015 Effect of Bt soybean on larvae of *Helicoverpa armigera* (Hübner) (Lepidoptera: Noctuidae). J. Agric. Sci. 7, 90 (10.5539/jas.v7n8p90)

[69] Yang Z, Chen H, Tang W, Hua HX, Lin YJ 2011 Development and characterisation of transgenic rice expressing two *Bacillus thuringiensis* genes. Pest Manag. Sci. 67, 414–422. (10.1002/ps.2079) 21394874

[70] Wang YN, Ke KQ, Li YH, Han LZ, Liu YM, Hua HX, Peng YF 2016 Comparison of three transgenic Bt rice lines for insecticidal protein expression and resistance against a target pest, *Chilo suppressalis* (Lepidoptera: Crambidae). Insect Sci. 23, 78–87. (10.1111/1744-7917.12178) 25284137

[71] High SM, Cohen MB, Shu QY, Altosaar I 2004 Achieving successful deployment of Bt rice. Trends Plant Sci. 9, 286–292. (10.1016/j.tplants.2004.04.002) 15165560

[72] Cheng X, Sardana R, Kaplan H, Altosaar I 1998 Agrobacterium-transformed rice plants expressing synthetic CryIA(b) and CryIA(c) genes are highly toxic to striped stem borer and yellow stem borer. Proc. Natl Acad. Sci. USA 95, 2767–2772. (10.1073/pnas.95.6.2767) 9501164 PMC19643

[73] Li ZY, Sui H, Xu YB, Han LZ, Chen FJ 2012 Effects of insect-resistant transgenic Bt rice with a fused Cry1Ab+Cry1Ac gene on population dynamics of the stem borers, *Chilo suppressalis* and *Sesamia inferens*, occurring in paddyfield. Acta Ecol. Sin. 32, 1783–1789. (10.5846/stxb201102260222)

[74] Huang F, Andow DA, Buschman LL 2011 Success of the high-dose/refuge resistance management strategy after 15 years of Bt crop use in North America. Entomol. Exp. Appl. 141, 1–16. (10.1111/j.1570-7458.2011.01190.x)

[75] Tabashnik BE et al. 2012 Sustained susceptibility of pink bollworm to Bt cotton in the United States. Gm Crops Food 3, 194–200. (10.4161/gmcr.20329) 22572905

[76] Bagla P 2010 Hardy cotton-munching pests are latest blow to GM crops. Science 327, 1439 (10.1126/science.327.5972.1439) 20299559

[77] Mohan KS, Ravi KC, Suresh PJ, Sumerford D, Head GP 2016 Field resistance to the *Bacillus thuringiensis* protein Cry1Ac expressed in Bollgard® hybrid cotton in pink bollworm, *Pectinophora gossypiella* (Saunders), populations in India. Pest Manag. Sci. 72, 738–746. (10.1002/ps.4047) 26016704

[78] Karihaloo JL, Kumar PA 2009 Bt cotton in India: a status report. Status Report 31, 2115–2127.

[79] Naik VC, Kumbhare S, Kranthi S, Satija U, Kranthi KR 2018 Field-evolved resistance of pink bollworm, *Pectinophora gossypiella* (Saunders) (Lepidoptera: Gelechiidae), to transgenic *Bacillus thuringiensis* (Bt) cotton expressing crystal 1Ac (Cry1Ac) and Cry2Ab in India. Pest Manag. Sci. 74, 2544–2554. (10.1002/ps.5038) 29697187

[80] Zhao JZ, Cao J, Collins HL, Bates SL, Roush RT, Earle ED, Shelton AM 2005 Concurrent use of transgenic plants expressing a single and two *Bacillus thuringiensis* genes speeds insect adaptation to pyramided plants. Proc. Natl Acad. Sci. USA 102, 8426–8430. (10.1073/pnas.0409324102) 15939892 PMC1150809

[81] Wan P, Huang YX, Wu HH, Huang MS, Cong SB, Tabashnik BE, Wu KM 2012 Increased frequency of pink bollworm resistance to Bt toxin Cry1Ac in China. PLoS ONE 7, e29975 (10.1371/journal.pone.0029975) 22238687 PMC3251611

[82] Wan P et al 2017 Hybridizing transgenic Bt cotton with non-Bt cotton counters resistance in pink bollworm. Proc. Natl Acad. Sci. USA 114, 5413–5418. (10.1073/pnas.1700396114) 28483999 PMC5448178

[83] Wu KM 2007 Monitoring and management strategy for *Helicoverpa armigera* resistance to Bt cotton in China. J. Invertebr. Pathol. 95, 220–223. (10.1016/j.jip.2007.03.012) 17467730

[84] Li YH, Gao YL, Wu KM 2017 Function and effectiveness of natural refuge in IRM strategies for Bt crops. Curr. Opin. Insect Sci. 21, 1–6. (10.1016/j.cois.2017.04.007) 28822481

[85] Jin L, Zhang HN, Lu YH, Yang YH, Wu KM, Tabashnik BE, Wu YD 2015 Large-scale test of the natural refuge strategy for delaying insect resistance to transgenic Bt crops. Nat. Biotechnol. 33, 169–174. (10.1038/nbt.3100) 25503384

[86] Blanco CA et al. 2016 Current situation of pests targeted by Bt crops in Latin America. Curr. Opin. Insect Sci. 15, 131–138. (10.1016/j.cois.2016.04.012) 27436743

[87] Fatoretto JC, Michel AP, Silva-Filho MC, Silva N 2017 Adaptive potential of fall armyworm (Lepidoptera: Noctuidae) limits Bt trait durability in Brazil. J. Int. Pest Manage. 8, 1–10. (10.1093/jipm/pmx011)

[88] Oppert B, Kramer KJ, Beeman RW, Johnson D, Mcgaughey WH 1997 Proteinase-mediated insect resistance to *Bacillus thuringiensis* toxins. J. Biol. Chem. 272, 23 473–23 476. (10.1074/jbc.272.38.23473) 9295279

[89] Herrero S, Ferré J, Escriche B 2001 Mannose phosphate isomerase isoenzymes in *Plutella xylostella* support common genetic bases of resistance to *Bacillus thuringiensis* toxins in lepidopteran species. Appl. Environ. Microbiol. 67, 979–981. (10.1128/AEM.67.2.979-981.2001) 11157273 PMC92677

[90] Forcada C, Alcácer E, Garcerá MD, Martínez R 1996 Differences in the midgut proteolytic activity of two *Heliothis virescens* strains, one susceptible and one resistant to *Bacillus thuringiensis* toxins. Arch Insect Biochem. 31, 257–272. (10.1002/(SICI)1520-6327)

[91] Li H, Oppert B, Higgins RA, Huang F, Zhu KY, Buschman LL 2004 Comparative analysis of proteinase activities of *Bacillus thuringiensis*-resistant and -susceptible *Ostrinia nubilalis* (Lepidoptera: Crambidae). Insect. Biochem. Mol. Biol. 34, 753–762. (10.1016/j.ibmb.2004.03.010) 15262280

[92] Liu CX, Xiao YT, Li XC, Oppert B, Tabashnik BE, Wu KM 2014 Cis-mediated down-regulation of a trypsin gene associated with Bt resistance in cotton bollworm. Sci. Rep. 4, 7219 (10.1038/srep07219) 25427690 PMC4245529

[93] Wei J et al. 2016 Activation of Bt protoxin Cry1Ac in resistant and susceptible cotton bollworm. PLoS ONE 11, e0156560 (10.1371/journal.pone.0156560) 27257885 PMC4892611

[94] Vellichirammal NN, Wang HC, Eyun SI, Moriyama EN, Coates BS, Miller NJ, Siegfried BD 2015 Transcriptional analysis of susceptible and resistant European corn borer strains and their response to Cry1F protoxin. BMC Genomics 16, 558 (10.1186/s12864-015-1751-6) 26220297 PMC4518661

[95] Tanaka S, Endo H, Adegawa S, Iizuka A, Imamura K, Kikuta S, Sato R 2017 *Bombyx mori* ABC transporter C2 structures responsible for the receptor function of *Bacillus thuringiensis* Cry1Aa toxin. Insect. Biochem. Mol. Biol. 91, 44–54. (10.1016/j.ibmb.2017.11.002) 29128667

[96] Qiu L, Wang P, Wu T, Li B, Wang X, Lei C, Lin Y, Zhao J, Ma W 2018 Downregulation of *Chilo suppressalis* alkaline phosphatase genes associated with resistance to three transgenic *Bacillus thuringiensis* rice lines. Insect. Mol. Biol. 27, 83–89. (10.1111/imb.12349) 28940938

[97] Xu XJ, Yu LY, Wu YD 2005 Disruption of a cadherin gene associated with resistance to Cry1Ac delta-endotoxin of *Bacillus thuringiensis* in *Helicoverpa armigera*. Appl. Environ. Microbiol. 71, 948–954. (10.1128/AEM.71.2.948-954.2005) 15691952 PMC546791

[98] Tay WT et al. 2015 Insect resistance to *Bacillus thuringiensis* toxin Cry2Ab is conferred by mutations in an ABC transporter subfamily A protein. PLoS Genet. 11, e1005534 (10.1371/journal.pgen.1005534) 26583651 PMC4652872

[99] Xiao YT, Zhang T, Liu CX, Heckel DG, Li XC, Tabashnik BE, Wu KM 2014 Mis-splicing of the ABCC2 gene linked with Bt toxin resistance in *Helicoverpa armigera*. Sci. Rep. 4, 6184 (10.1038/srep06184) 25154974 PMC4143771

[100] Zhang SP, Cheng HM, Gao YL, Wang GR, Liang GM, Wu KM 2009 Mutation of an aminopeptidase N gene is associated with *Helicoverpa armigera* resistance to *Bacillus thuringiensis* Cry1Ac toxin. Insect. Biochem. Mol. Biol. 39, 421–429. (10.1016/j.ibmb.2009.04.003) 19376227

[101] Rajagopal R, Arora N, Sivakumar S, Rao NG, Nimbalkar SA, Bhatnagar RK 2009 Resistance of *Helicoverpa armigera* to Cry1Ac toxin from *Bacillus thuringiensis* is due to improper processing of the protoxin. Biochem. J. 419, 309–316. (10.1042/BJ20081152) 19146482

[102] Xiao YT et al. 2017 A single point mutation resulting in cadherin mislocalization underpins resistance against *Bacillus thuringiensis* toxin in cotton bollworm. J. Biol. Chem. 292, 2933–2943. (10.1074/jbc.M116.768671) 28082675 PMC5314188

[103] Ning CM, Wu KM, Liu CX, Gao YL, Juratfuentes JL, Gao XW 2010 Characterization of a Cry1Ac toxin-binding alkaline phosphatase in the midgut from *Helicoverpa armigera* (Hübner) larvae. J. Insect. Physiol. 56, 666–672. (10.1016/j.jinsphys.2010.02.003) 20170658

[104] Gahan LJ, Gould F, Heckel DG 2001 Identification of a gene associated with Bt resistance in *Heliothis virescens*. Science 293, 857–860. (10.1126/science.1060949) 11486086

[105] Xie R, Zhuang M, Ross LS, Gomez I, Oltean DI, Bravo A, Soberon M, Gill SS 2005 Single amino acid mutations in the cadherin receptor from *Heliothis virescens* affect its toxin binding ability to Cry1A toxins. J. Biol. Chem. 280, 8416–8425. (10.1074/jbc.M408403200) 15572369

[106] Jurat-Fuentes JL, Adang MJ 2004 Characterization of a Cry1Ac-receptor alkaline phosphatase in susceptible and resistant *Heliothis virescens* larvae. FEBS J. 271, 3127–3135. (10.1111/j.1432-1033.2004.04238.x) 15265032

[107] Gahan LJ, Pauchet Y, Vogel H, Heckel DG 2010 An ABC transporter mutation is correlated with insect resistance to *Bacillus thuringiensis* Cry1Ac toxin. PLoS Genet. 6, e1001248 (10.1371/journal.pgen.1001248) 21187898 PMC3002984

[108] Zhang Z, Teng XL, Ma WH, Li F 2017 Knockdown of two cadherin genes confers resistance to Cry2A and Cry1C in *Chilo suppressalis*. Sci. Rep. 7, 5992 (10.1038/s41598-017-05110-9) 28729614 PMC5519675

[109] Jin TT, Chang X, Gatehouse AMR, Wang ZY, Edwards MG, He KL 2014 Downregulation and mutation of a cadherin gene associated with Cry1Ac resistance in the Asian corn borer, *Ostrinia furnacalis* (Guenée). Toxins 6, 2676–2693. (10.3390/toxins6092676) 25216082 PMC4179154

[110] Bel Y, Siqueira HA, Siegfried BD, Ferré J, Escriche B 2009 Variability in the cadherin gene in an *Ostrinia nubilalis* strain selected for Cry1Ab resistance. Insect. Biochem. Mol. Biol. 39, 218–223. (10.1016/j.ibmb.2008.11.005) 19114103

[111] Coates BS, Siegfried BD 2015 Linkage of an ABCC transporter to a single qtl that controls *Ostrinia nubilalis*, larval resistance to the *Bacillus thuringiensis*, Cry1Fa toxin. Insect. Biochem. Mol. Biol. 63, 86–96. (10.1016/j.ibmb.2015.06.003) 26093031

[112] Coates BS, Sumerford DV, Siegfried BD, Hellmich RL, Abel CA 2013 Unlinked genetic loci control the reduced transcription of aminopeptidase N 1 and 3 in the European corn borer and determine tolerance to *Bacillus thuringiensis* Cry1Ab toxin. Insect. Biochem. Mol. Biol. 43, 1152–1160. (10.1016/j.ibmb.2013.09.003) 24121099

[113] Khajuria C, Buschman LL, Chen M-S, Siegfried BD, Zhu KY 2011 Identification of a novel aminopeptidase p-like gene (onapp) possibly involved in Bt toxicity and resistance in a major corn pest (*Ostrinia nubilalis*). PLoS ONE 6, e23983 (10.1371/journal.pone.0023983) 21887358 PMC3161092

[114] Morin S et al. 2003 Three cadherin alleles associated with resistance to *Bacillus thuringiensis* in pink bollworm. Proc. Natl Acad. Sci. USA 100, 5004–5009. (10.1073/pnas.0831036100) 12695565 PMC154288

[115] Wang L et al. 2018 Resistance to *Bacillus thuringiensis* linked with a cadherin transmembrane mutation affecting cellular trafficking in pink bollworm from China. Insect. Biochem. Mol. Biol. 94, 28–35. (10.1016/j.ibmb.2018.01.004) 29408651

[116] Fabrick JA, Ponnuraj J, Singh A, Tanwar RK, Unnithan GC, Yelich AJ, Li X, Carrière Y, Tabashnik BE 2014 Alternative splicing and highly variable cadherin transcripts associated with field-evolved resistance of pink bollworm to Bt cotton in India. PLoS ONE 9, e97900 (10.1371/journal.pone.0097900) 24840729 PMC4026531

[117] Fabrick JA, Mathew LG, Tabashnik BE, Li X 2011 Insertion of an intact CR1 retrotransposon in a cadherin gene linked with Bt resistance in the pink bollworm, *Pectinophora gossypiella*. Insect. Mol. Biol. 20, 651–665. (10.1111/j.1365-2583.2011.01095.x) 21815956

[118] Guo ZJ et al. 2015 MAPK signaling pathway alters expression of midgut ALP and ABCC genes and causes resistance to *Bacillus thuringiensis* Cry1Ac toxin in diamondback moth. PLoS Genet. 11, e1005124 (10.1371/journal.pgen.1005124) 25875245 PMC4395465

[119] Baxter SW, Badenes-Pérez FR, Morrison A, Vogel H, Crickmore N, Kain W, Wang P, Heckel DG, Jiggins CD 2011 Parallel evolution of *Bacillus thuringiensis* toxin resistance in Lepidoptera. Genetics 189, 675–679. (10.1534/genetics.111.130971) 21840855 PMC3189815

[120] Yuan XD, Zhao M, Wei JZ, Zhang WN, Wang BJ, Khaing KM, Liang GM 2017 New insights on the role of alkaline phosphatase 2 from *Spodoptera exigua* (Hübner) in the action mechanism of Bt toxin Cry2Aa. J. Insect. Physiol. 98, 101–107. (10.1016/j.jinsphys.2016.12.004) 28034678

[121] Herrero S, Gechev T, Bakker PL, Moar WJ, Maagd RAD 2005 *Bacillus thuringiensis*, Cry1Ca-resistant *Spodoptera exigua*, lacks expression of one of four aminopeptidase N genes. BMC Genomics 6, 96 (10.1186/1471-2164-6-96) 15978131 PMC1184072

[122] Park Y et al. 2014 ABCC transporters mediate insect resistance to multiple Bt toxins revealed by bulk segregant analysis. BMC Biol. 12, 46 (10.1186/1741-7007-12-46) 24912445 PMC4071345

[123] Jakka SR, Gong L, Hasler J, Banerjee R, Sheets JJ, Narva K, Blanco CA, Jurat-Fuentes JL 2015 Field-evolved mode 1 fall armyworm resistance to Bt corn associated with reduced Cry1Fa toxin binding and midgut alkaline phosphatase expression. Appl. Environ. Microbiol. 82, 1023–1034. (10.1128/AEM.02871-15) 26637593 PMC4751857

[124] Tiewsiri K, Wang P 2011 Differential alteration of two aminopeptidases N associated with resistance to *Bacillus thuringiensis* toxin Cry1Ac in cabbage looper. Proc. Natl Acad. Sci. USA 108, 14 037–14 042. (10.1073/pnas.1102555108) PMC3161562 21844358

[125] Zhao J, Jin L, Yang YH, Wu YD 2010 Diverse cadherin mutations conferring resistance to *Bacillus thuringiensis* toxin Cry1Ac in *Helicoverpa armigera*. Insect. Biochem. Mol. Biol. 40, 113–118. (10.1016/j.ibmb.2010.01.001) 20079435

[126] Li J et al. 2017 FOXA transcriptional factor modulates insect susceptibility to *Bacillus thuringiensis* Cry1Ac toxin by regulating the expression of toxin-receptor ABCC2 and ABCC3 genes. Insect. Biochem. Mol. Biol. 88, 1–11. (10.1016/j.ibmb.2017.07.004) 28736301

[127] Xiao YT, Liu KY, Zhang DD, Gong LL, He F, Soberón M, Bravo A, Tabashnik BE, Wu KM 2016 Resistance to *Bacillus thuringiensis* mediated by an ABC transporter mutation increases susceptibility to toxins from other bacteria in an invasive insect. PLoS Pathog. 12, e1005450 (10.1371/journal.ppat.1005450) 26872031 PMC4752494

[128] Wang J, Wang HD, Liu SY, Liu LP, Tay WT, Walsh TK, Yang YH, Wu YD 2017 CRISPR/Cas9 mediated genome editing of *Helicoverpa armigera* with mutations of an ABC transporter gene HaABCA2 confers resistance to *Bacillus thuringiensis* Cry2A toxins. Insect. Biochem. Mol. Biol. 87, 147 (10.1016/j.ibmb.2017.07.002) 28705634

[129] Guo ZJ, Kang S, Zhu X, Xia JX, Wu QJ, Wang SL, Xie W, Zhang YJ 2015 Down-regulation of a novel ABC transporter gene (Pxwhite) is associated with Cry1Ac resistance in the diamondback moth, *Plutella xylostella* (L.). Insect. Biochem. Mol. Biol. 59, 30–40. (10.1016/j.ibmb.2015.01.009) 25636859

[130] Jurat-Fuentes JL et al. 2011 Reduced levels of membrane-bound alkaline phosphatase are common to lepidopteran strains resistant to cry toxins from *Bacillus thuringiensis*. PLoS ONE 6, e17606 (10.1371/journal.pone.0017606) 21390253 PMC3046977

[131] Sivakumar S, Rajagopal R, Venkatesh GR, Srivastava A, Bhatnagar RK 2007 Knockdown of aminopeptidase-N from *Helicoverpa armigera* larvae and in transfected Sf21 cells by RNA interference reveals its functional interaction with *Bacillus thuringiensis* insecticidal protein Cry1Ac. J. Biol. Chem. 282, 7312–7319. (10.1074/jbc.M607442200) 17213205

[132] Xu L, Wang Z, Zhang J, Ferry N, Edwards MG, Gatehouse AM, He K 2014 Characterization of four midgut aminopeptidase N isozymes from *Ostrinia furnacalis* strains with different susceptibilities to *Bacillus thuringiensis*. J. Invertebr. Pathol. 115, 95–98. (10.1016/j.jip.2013.11.001) 24269376

[133] Ren XL, Ma Y, Cui JJ, Li GQ 2014 RNA interference-mediated knockdown of three putative aminopeptidases N affects susceptibility of *Spodoptera exigua* larvae to *Bacillus thuringiensis* Cry1Ca. J. Insect. Physiol. 67, 28–36. (10.1016/j.jinsphys.2014.06.002) 24932922

[134] Contreras E, Schoppmeier M, Real MD, Rausell C 2013 Sodium solute symporter and cadherin proteins act as *Bacillus thuringiensis* Cry3Ba toxin functional receptors in *Tribolium castaneum*. J. Biol. Chem. 288, 18 013–18 021. (10.1074/jbc.M113.474445) PMC3689946 23645668

[135] Griffitts JS et al. 2005 Glycolipids as receptors for *Bacillus thuringiensis* crystal toxin. Science 307, 922–925. (10.1126/science.1104444) 15705852

[136] Kumaraswami NS, Maruyama T, Kurabe S, Kishimoto T, Mitsui T, Hori H 2001 Lipids of brush border membrane vesicles (BBMV) from *Plutella xylostella* resistant and susceptible to Cry1Ac delta-endotoxin of *Bacillus thuringiensis*. Comp. Biochem. Phys. B 129, 173–183. (10.1016/S1096-4959(01)00327-X) 11337261

[137] Chen YZ, Huang YP, Liu Q, Xu J, Hogenhout S, Huang YP, Tan AJ 2017 Fishing for new Bt receptors in diamondback moth. bioRxiv. (10.1101/181834)

[138] Ding SY, Li HY, Li XF, Zhang ZY 2001 Effects of Bt transgenic poplar on detoxification enzyme and ache in American white moth larvae. J. Northeast For. Univ. 29, 28–30.

[139] Shi MJ, Lu PL, Shi XL, Yang YZ 2011 Effect of insect-resistant transgenic maize on growth and development, utilization of nutrients and *in vivo* activity of the detoxification enzymes of the Asian corn borer, *Ostrinia furnacalis* (Lepidoptera: Pyralidae). Eur. J. Entomol. 108, 547–552. (10.14411/eje.2011.070)

[140] Gunning RV, Dang HT, Kemp FC, Nicholson IC, Moores GD 2005 New resistance mechanism in *Helicoverpa armigera* threatens transgenic crops expressing *Bacillus thuringiensis* Cry1Ac toxin. Appl. Environ. Microbiol. 71, 2558–2563. (10.1128/AEM.71.5.2558-2563.2005) 15870346 PMC1087549

[141] Xu PJ, Liu YQ, Graham RI, Wilson K, Wu KM 2014 Densovirus is a mutualistic symbiont of a global crop pest (*Helicoverpa armigera*) and protects against a baculovirus and Bt biopesticide. PLoS Pathog. 10, e1004490 (10.1371/journal.ppat.1004490) 25357125 PMC4214819

[142] Chougule NP, Li H, Liu S, Linz LB, Narva KE, Meade T, Bonning BC 2013 Retargeting of the *Bacillus thuringiensis* toxin Cyt2Aa against hemipteran insect pests. Proc. Natl Acad Sci. USA. 110, 8465 (10.1073/pnas.1222144110) 23650347 PMC3666667

[143] Goergen G, Kumar PL, Sankung SB, Togola A, Tamò M 2016 First report of outbreaks of the fall armyworm *Spodoptera frugiperda* J E Smith) (Lepidoptera, Noctuidae), a new alien invasive pest in West and Central Africa. PLoS ONE 11, e0165632 (10.1371/journal.pone.0165632) 27788251 PMC5082806

[144] Stokstad E 2017 New crop pest takes Africa at lightning speed. Science 356, 473–474. (10.1126/science.356.6337.473) 28473543

[145] Yu XD, Liu ZC, Huang SL, Chen ZQ, Sun YW, Duan PF, Ma YZ, Xia LQ 2016 RNAi-mediated plant protection against aphids. Pest Manag. Sci. 72, 1090–1098. (10.1002/ps.4258) 26888776

[146] Zotti M, Santos EAD, Cagliari D, Christiaens O, Taning CNT, Smagghe G 2018 RNA interference technology in crop protection against arthropod pests, pathogens and nematodes. Pest Manag. Sci. 74, 1239–1250. (10.1002/ps.4813) 29194942

[147] Ni M et al. 2017 Next-generation transgenic cotton: pyramiding rnai and Bt counters insect resistance. Plant Biotechnol. J. 15, 1204–1213. (10.1111/pbi.12709) 28199783 PMC5552478

[148] Garczynski SF, Martin JA, Griset M, Willett LS, Cooper WR, Swisher KD, Unruh TR 2017 CRISPR/Cas9 editing of the codling moth (Lepidoptera: Tortricidae) CpomOR1 gene affects egg production and viability. J. Econ. Entomol. 110, 1847–1855. (10.1093/jee/tox166) 28854653

[149] Karaminejadranjbar M, Eckermann KN, Ahmed HMM, Sánchez CHM, Dippel S, Marshall JM, Wimmer EA 2018 Consequences of resistance evolution in a Cas9-based sex conversion-suppression gene drive for insect pest management. Proc. Natl Acad. Sci. USA 115, 6189–6194. (10.1073/pnas.1713825115) 29844184 PMC6004448

[150] Pauchet Y, Bretschneider A, Augustin S, Heckel D 2016 A P-glycoprotein is linked to resistance to the *Bacillus thuringiensis* Cry3Aa toxin in a leaf beetle. Toxins 8, 362 (10.3390/toxins8120362) PMC5198556 27929397

